# Dataset on the agronomic characteristics and combining ability of new parental lines in the two-line hybrid rice systems in Vietnam

**DOI:** 10.1016/j.dib.2021.107069

**Published:** 2021-04-20

**Authors:** Quang Van Tran, Long Thien Tran, Dung Thi Kim Nguyen, Linh Hong Ta, Loc Van Nguyen, Tuan Thanh Nguyen

**Affiliations:** a Faculty of Agronomy, Vietnam National University of Agriculture, Hanoi, Vietnam; b Crops Research and Development Institute, Vietnam National University of Agriculture, Hanoi, Vietnam; c Vietnam Academy of Agriculture Sciences, Hanoi, Vietnam

**Keywords:** Combining ability analysis, Rice TGMS lines, Two line hybrid rice

## Abstract

This article provides a dataset on the analysis of morphological characteristics and combining ability of some parental lines in the two line hybrid rice system in Vietnam. Four thermo-sensitive male sterile lines and seven pollen restorer lines were used in a Line x Tester mating system to produce twenty-eight hybrids. The parental lines were characterized on 14 agronomic traits in a completely random design experiment. The 28 hybrids were evaluated on 10 traits related to grain yield and morphology in a randomized complete block design experiment with three replications. A line x tester analysis was conducted to estimate the combining ability, genetic variance, and the contribution of parental lines to genetic variation in hybrids. This dataset is valuable for rice breeders in subtropical countries to orient the strategy for breeding of hybrid rice varieties with high efficiency.

## Specification Table

**Table:** 

Subject	Agriculture
Specific subject area	Hybrid breeding, Self-pollinated crops
Type of data	Table, Figure
How data were acquired	Line x Tester analysis
Data format	Raw
Parameters for data collection	Research materials included eleven parental lines (4 TGMS lines and 7 pollen restorer lines) and their 28 hybrids. The parental lines were grown in winter-spring 2017- 2018, the hybrids were grown in summer-autumn 2018.
Description of data collection	Total of 14 agronomic characteristics were evaluated on parental lines; the hybrids were monitored on 10 traits related to grain yield and morphology to conduct a Line x Tester analysis. The data collection method was in accordance with standards suggested by International Rice Research Institute.
Data source location	Institution: Vietnam National University of Agriculture City/Town/Region: Gia Lam, Ha Noi Country: Vietnam
Data accessibility	With the article

## Value of Data

• Evaluation of combining ability is mostly used for selection of potential parental lines for breeding new hybrid rice varieties.
• The dataset shows the combining ability of new parental rice lines on important characteristics related to grain yield and morphology. Moreover, the data on parental lines will be a valuable reference to establish a model of TGMS line for the two-line hybrid rice system in Vietnam.
• The Line x Tester analysis described in details could be applicable for hybrid breeding in other self-pollinated crops in Vietnam.

## 1 Data Description

Fig. 1 presents the micro-climate data for temperature and humidity during the period from January 2018 to December 2018. Fig. 2 shows the typical phenotypes of parental lines.

Eleven parental lines were characterized on 14 agronomic characteristics, including plant growth and morphological characters (Table 1), grain yield-related traits (Table 2) and some special characteristics of female parent lines (TGMS) (Table 3).

Twenty-eight hybrids were evaluated on 10 characteristics, including number of panicle per plant, number of filled grain per panicle, proportion of filled grain, 1000-grain weight, grain yield, proportion of husked grain, proportion of milled grain, proportion of whole grain, white grain length and white grain width. The data were collected to analyze the variance of combining ability (Table 4), general combining ability of parental lines (Table 5), specific combining ability between male (line) and female (tester) parental lines (Tables 6 and 7) and the contribution of parents towards genetic variance among hybrids (Table 8).

A list of parental lines with information of their origin was presented on Table 9. Table 10 described in detail of data collection method, based on the standard of International Rice Research Institute [1].

### 1.1 Micro-climate data during the experimental period

**Fig. 1:**
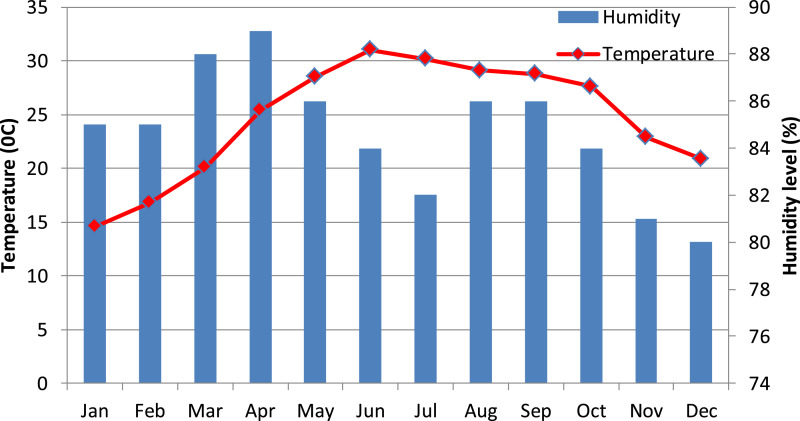
Air temperature and humidity in 2018.

### 1.2 Agronomic characteristics of parental lines

**Table 1:** Plant growth and morphological characteristics of parental lines in Spring 2018.

	Maturity	Plant	Culm	Leaf blade	Flag leaf	Brown rice
Line	(day)	height (cm)	angle	color	angle	shape
DG18	125	100	3	Green	5	3
ND502	123	92	1	Green	5	3
BT14	135	100	3	Green	5	3
D16	123	103	5	Green	5	3
G15–1	123	96	3	Dark green	5	3
13×17–1	122	90	1	Green	5	5
R23	125	94	1	Dark green	5	5
E15S	142	86	3	Purple margins	1	3
E16S	142	78	3	Green	1	3
E26S	155	78	3	Green	1	3
E30S	155	75	3	Green	1	3

**Table 2:** Grain yield and yield-related traits of parental lines in Spring 2018.

	Number of	Number of	Proportion of	1000-grain
Line	panicles/plant	grains/panicle	filled grain (%)	weight (g)
DG18	8.0	291.0	83.6	23.6
ND502	6.0	251.7	78.4	25.3
BT14	5.3	252.1	80.0	25.3
D16	6.4	258.0	80.3	25.5
G15–1	8.7	194.4	80.6	26.4
13×17–1	8.1	214.2	83.3	26.4
R23	10.1	177.5	86.5	25.6
E15S	9.7	74.1	63.3	24.6
E16S	9.7	80.4	82.5	23.8
E26S	7.5	86.4	77.4	23.2
E30S	7.2	87.6	62.6	24.0

**Table 3:** Male sterile- related traits of female parental lines in Summer 2018.

	Pollen sterility	Male sterility group	Stigma exsertion		
Line			One side	Both side	Score
E15S	3	TGMS	27.8	52.4	1
E16S	3	TGMS	26.5	52.7	1
E26S	3	TGMS	25.1	51.3	1
E30S	3	TGMS	28.5	49.6	1

**Fig. 2:**
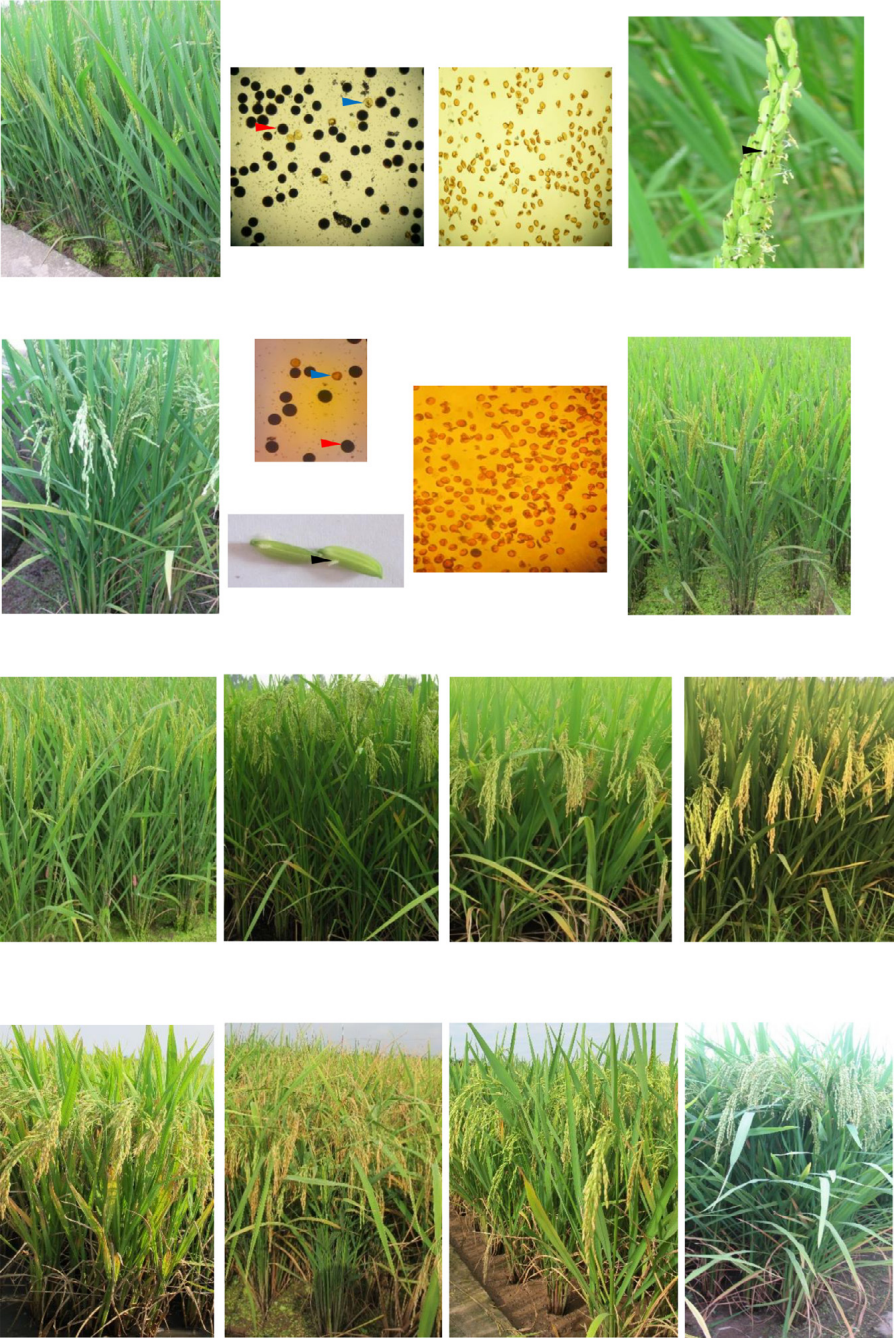
Phenotype of parental lines. The images of whole plants and fertile pollen were recorded in Spring 2018 while the flower and sterile pollen were observed in Summer 2018. In the pollen images, the red and blue arrows indicating the fertile and sterile pollen, respectively; in the flower images, the black arrow pointing the stigma exsertion.

### 1.3 Combining ability analysis

**Table 4:** Analysis of variance for combining ability in rice.

Source of variance	Degree of freedom	Number of panicle/ plant	Number of filled grain/ panicle	Proportion of filled grain	1000-grain weight	Grain yield	Proportion of husked grain	Proportion of milled grain	Proportion of whole grain	White grain length	White grain width
Replication	2	0.066	112.09	5.17	0.01	13.41	1.32	5.62	16.43	0.11	0.00

Crosses	27	116.03	92,068.12	4915.60	462.66	8388.96	654.51	543.20	10,530.60	11.12	1.44

GCA Line	6	37.64 **	60,182.16 ***	3086.81 ***	176.01 ns	3048.86 **	244.20 *	235.55 ***	1528.51 ns	2.88 ns	0.24 ns

GCA Tester	3	38.66 ***	6297.90 ns	341.29 ns	7.17 ns	1599.07 *	59.82 ns	132.71 ***	2570.76 *	2.56 *	0.04 ns

SCA Line*Tester	18	39.73 ***	25,588.06 ***	1487.50 ***	279.49 ***	3741.03 ***	350.49 ***	174.94 ***	6431.33 ***	5.68 ***	1.16 ***

Error	54	5.72	1698.41	135.76	4.27	151.66	90.76	65.10	205.67	0.46	0.01

σ^2^GCA		10.76	12,963.16	650.89	36.78	790.00	28.80	44.35	567.28	0.77	0.05

σ^2^SCA		11.34	7963.22	450.58	91.74	1196.46	86.58	36.61	2075.22	1.74	0.38

σ^2^GCA/σ^2^SCA		0.95	1.63	1.44	0.40	0.66	0.33	1.21	0.27	0.44	0.13

h^2^ (%)		65.50	76.50	74.29	44.50	56.91	39.95	70.78	35.35	46.90	20.89

GCA: General combining ability; SCA: Specific combining ability; σ^2^: Variance; h^2^: Narrow sense heritability; ns: non-significant; *, **, and ***: significant at P *≤* *0.1, P* *≤* *0.05, and P* *≤* *0.01, respectively.*

**Table 5:** Analysis of general combining ability of parental lines for 10 investigated characteristics.

Parental line		Number of panicle/ plant	Number of filled grain/ panicle	Proportion of filled grain	1000-grain weight	Grain yield	Proportion of husked grain	Proportion of milled grain	Proportion of whole grain	White grain length	White grain width
Male (Line)	DG18	0.301**	−31.724***	−1.786**	0.314	−8.111**	0.207ns	0.098ns	−3.192	0.177	−0.057
	ND502	0.043ns	−5.099**	3.273***	0.364	1.514**	−1.285*	−1.886***	−2.825	0.202	−0.030
	BT14	−0.715**	19.976***	2.098***	−1.961	−8.144**	−0.285ns	−0.869*	7.483	−0.098	−0.008
	D16	−1.115**	51.560***	1.198**	−2.169	−3.244**	1.215*	0.781*	−3.825	−0.174	0.070
	G15–1	0.210**	−6.749**	3.739***	1.423	6.398**	2.215*	2.131***	1.125	−0.208	0.093
	13×7–1	0.176*	1.651ns	5.414***	0.064	5.056**	1.207*	2.123***	4.892	−0.148	−0.035
	R23	1.101ns	−29.615***	−13.936***	1.964	6.531**	−3.276*	−2.377***	−3.658	0.251	−0.034
	*Error*	0.069	*1.619*	*0.458*	*0.081*	*0.484*	*0.374*	*0.317*	*0.563*	*0.026*	*0.005*
	LSD_0_*_,1_*	0.141	*3.297*	*0.933*	*ns*	*0.986*	*0.762*	*0.646*	*ns*	*ns*	*ns*
	*LSD_0,05_*	0.190	*4.449*	*1.259*	*ns*	*1.330*	*ns*	*0.871*	*ns*	*ns*	*ns*
	*LSD_0,01_*	ns	*7.196*	*2.036*	*ns*	*ns*	*ns*	*1.409*	*ns*	*ns*	*ns*

Female (Tester)	E15S	−0.693***	−1.550	−1.438	0.490	−5.380*	−0.932	−1.500**	−2.768*	0.019ns	−0.020
	E16S	−0.621***	11.602	−2.495	−0.100	−2.799*	−0.361	−0.738*	−7.682*	0.208*	−0.007
	E26S	0.421**	2.469	2.300	−0.281	2.368*	−0.065	0.405ns	4.470*	−0.249*	−0.007
	E30S	0.893***	−12.521	1.633	−0.110	5.811*	1.358	1.833***	5.980*	−0.068*	0.035
	*Error*	*0.071*	*3.363*	*0.951*	*0.168*	*1.421*	*0.778*	*0.659*	*1.171*	*0.055*	*0.008*
	*LSD_0,1_*	*0.164*	*ns*	*ns*	*ns*	*1.198*	*ns*	*0.556*	*0.987*	*0.046*	*ns*
	*LSD_0,05_*	*0.236*	*ns*	*ns*	*ns*	*ns*	*ns*	*0.799*	*ns*	*ns*	*ns*
	*LSD_0,01_*	*0.456*	*ns*	*ns*	*ns*	*ns*	*ns*	*1.541*	*ns*	*ns*	*ns*

*LSD: Least significant difference (at P* *≤* 0.1/0.05/0.01); ns: non-significant; *, **, and ***: significant at P *≤* *0.1, P* *≤* *0.05, and P* *≤* *0.01, respectively.*

**Table 6:** Specific combining ability for yield related characteristics.

TT	Hybrid combination	Number of panicle/ plant	Number of filled grain/ panicle	Proportion of filled grain	1000-grain weight	Grain yield
1	E15S/DG18	0.418*	9.535**	8.338***	−1.290***	4.430***
2	E16S/ DG18	−0.854***	−5.252ns	2.095*	0.100ns	−9.518***
3	E26S/ DG18	0.837***	−27.119***	−9.667***	0.881***	−0.185ns
4	E30S/ DG18	−0.401*	22.838***	−0.767ns	0.310*	5.273***
5	E15S/ND502	−0.024ns	−8.825**	1.113ns	−1.340***	−9.395***
6	E16S/ND502	−0.362*	8.023**	0.737ns	0.650***	2.257*
7	E26S/ND502	−0.705***	5.556ns	−1.658ns	−1.069***	−5.510***
8	E30S/ND502	1.090***	−4.754ns	−0.192ns	1.760***	12.648***
9	E15S/BT14	−0.099ns	−5.900ns	−7.645***	0.485**	−0.204ns
10	E16S/BT14	−0.637**	25.448***	3.512***	0.375*	1.715ns
11	E26S/BT14	1.187***	−20.319***	2.583**	−0.044ns	2.549**
12	E30S/BT14	−0.451*	0.771ns	1.550ns	−0.815***	−4.061***
13	E15S/D16	0.168ns	−25.183***	2.955**	0.326*	−3.404**
14	E16S/D16	−0.104ns	−4.636ns	1.545ns	0.383*	−2.551**
15	E26S/D16	−1.513***	55.631***	−1.383ns	−0.402**	−3.951***
16	E30S/D16	1.449***	−25.812***	−3.117**	−0.307*	9.906***
17	E15S/G15–1	−0.357*	11.425**	3.313***	1.301***	3.555***
18	E16S/G15–1	1.005***	−4.327ns	−1.230ns	−4.608***	−6.126***
19	E26S/G15–1	−0.305ns	−19.794***	−0.325ns	4.273***	4.707***
20	E30S/G15–1	−0.343ns	12.696***	−1.758*	−0.965***	−2.136*
21	E15S/13×17–1	−0.357*	17.025***	0.438ns	−2.240***	−5.904***
22	E16S/13×17–1	0.371*	−6.794ns	−1.905*	1.250***	6.482***
23	E26S/13×17–1	0.462**	2.973ns	0.900ns	−1.869***	3.715***
24	E30S/13×17–1	−0.476**	−13.204***	0.567ns	2.860***	−4.294***
25	E15S/R23	0.251ns	1.925ns	−8.512***	2.760***	10.921***
26	E16S/R23	0.580**	−12.461***	−4.755***	1.850***	7.740***
27	E26S/R23	0.037ns	3.073ns	9.550***	−1.769***	−1.326ns
28	E30S/R23	−0.868***	7.463*	3.717***	−2.840***	−17.336***
*Error*		*0.188*	*3.238*	*0.915*	*0.162*	*0.968*
LSD_0,1_		*0.354*	*6.090*	*1.721*	*0.305*	*1.821*
LSD_0,05_		*0.461*	*7.940*	*2.244*	*0.397*	*2.374*
*LSD_0,01_*		*0.679*	*11.686*	*3.302*	*0.585*	*3.494*

*LSD: Least significant difference (at P* *≤* 0.1/0.05/0.01); ns: non-significant; *, **, and ***: significant at P *≤* *0.1, P* *≤* *0.05, and P* *≤* *0.01, respectively.*

**Table 7:** Specific combining ability for grain morphology.

TT	Hybrid combination	Proportion of husked grain	Proportion of milled grain	Proportion of whole grain	White grain length	White grain width
1	E15S/DG18	0.940ns	1.483*	18.235***	−0.035ns	0.074***
2	E16S/ DG18	0.336ns	0.755ns	−6.351***	−0.534***	−0.043***
3	E26S/ DG18	0.074ns	−0.388ns	−6.704***	0.426***	−0.143***
4	E30S/ DG18	−1.350ns	−1.850**	−5.180***	0.142**	0.112***
5	E15S/ND502	−5.568***	−2.500***	−16.132***	−0.156**	0.046***
6	E16S/ND502	1.861**	−1.262*	−9.618***	0.244***	−0.070***
7	E26S/ND502	1.565*	1.595**	4.730***	−0.302***	0.033***
8	E30S/ND502	2.142**	2.167**	21.020***	0.214***	−0.009ns
9	E15S/BT14	1.432*	0.483ns	10.060***	0.044ns	−0.079***
10	E16S/BT14	0.861ns	−0.245ns	−0.226ns	0.341***	0.008ns
11	E26S/BT14	−1.435*	−1.388*	−6.245***	−0.102*	0.008ns
12	E30S/BT14	−0.858ns	1.150ns	−3.588**	−0.283***	0.063***
13	E15S/D16	1.932**	−0.467ns	−8.565***	−0.284***	−0.153***
14	E16S/D16	−4.639***	−1.929**	8.082***	0.417***	−0.070***
15	E26S/D16	1.065ns	0.929ns	0.596ns	−0.026ns	0.131***
16	E30S/D16	1.642*	1.467*	−0.113ns	−0.107*	0.092***
17	E15S/G15–1	0.932ns	−0.483ns	2.118*	−0.150**	0.120***
18	E16S/G15–1	−1.639*	−1.279*	−4.768***	−0.250***	−0.093***
19	E26S/G15–1	0.065ns	1.579**	−0.720ns	0.307***	0.107***
20	E30S/G15–1	0.642ns	0.183ns	3.370**	0.093ns	−0.135***
21	E15S/13×17–1	1.907**	1.492*	4.885***	0.190**	0.045***
22	E16S/13×17–1	−0.631ns	0.730**	6.965***	−0.309***	−0.065***
23	E26S/13×17–1	1.074ns	−0.413ns	−5.154***	0.148**	0.132***
24	E30S/13×17–1	−2.350**	−1.808**	−6.696***	−0.029ns	−0.111***
25	E15S/R23	−1.576*	−0.008ns	−10.599***	0.391***	−0.053***
26	E16S/R23	3.852***	3.230***	5.915***	0.092ns	0.334***
27	E26S/R23	−2.410**	−1.913**	13.496***	−0.451***	−0.269***
28	E30S/R23	0.133	−1.308*	−8.813***	−0.032ns	−0.012
*Error*		*0.748*	*0.634*	*1.127*	*0.053*	*0.009*
LSD_0,1_		*1.407*	*1.192*	*2.120*	*0.100*	*0.017*
LSD_0,05_		*1.834*	*1.555*	*2.764*	*0.130*	*0.022*
*LSD_0,01_*		*2.700*	*2.288*	*4.067*	*0.191*	*0.032*

*LSD: Least significant difference (at P* *≤* 0.1/0.05/0.01); ns: non-significant; *, **, and ***: significant at P *≤* *0.1, P* *≤* *0.05, and P* *≤* *0.01, respectively.*

**Table 8:** Contribution of male, female and male x female interactions to the variance of investigated characters in hybrids.

Traits	Percent contribution of		
	Male	Female	Male x Female
Number of panicle/ plant	33.44	33.32	34.24
Number of filled grain/ panicle	65.37	6.84	27.79
Proportion of filled grain	62.80	6.94	30.26
1000-grain weight	38.04	1.55	60.41
Grain yield	36.34	19.06	44.60
Proportion of husked grain	37.31	9.14	53.55
Proportion of milled grain	43.36	24.43	32.21
Proportion of whole grain	14.52	24.41	61.07
White grain length	25.90	23.01	51.09
White grain width	16.67	2.53	80.80

**Table 9:** The name and origin of parental lines.

Line	Origin
DG18	Selected from segregated population of a three-line hybrid rice cultivar “Xuyên Hương 178″, origins from China
ND502	Selected from a local rice cultivar “Séng cù”, Lao Cai province, Vietnam
BT14	Pure line imported from China
D16	Selected from a hybrid AG1 x R998 of An Giang plant protection company, Vietnam
G15–1	Selected from a hybrid BC15 x HV3; in which, BC15 is a pure line of Thai Binh seed company, Vietnam; HV3 is a pure line named “Hương Việt 3″ of Vietnam National University of Agriculture, Vietnam
13×17–1	A local rice cultivar selected from Dien Bien, Vietnam in spring 2017
R23	Selected from a hybrid R3 x KH116; in which, R3 is the male parental line of a two-line hybrid rice variety “HT3–3″ of VNUA, Vietnam; KH116 is a pure line imported from China
E15S	Selected from a hybrid 135S x “Hoa sữa”; in which, 135S is a TGMS lines of VNUA, Vietnam; “Hoa sữa” is a pure line imported from USA
E16S	Selected from a hybrid 135S x SH6; in which, SH6 is a pure line of Field Crops Research Institute, Hai Duong province, Vietnam
E26S	Selected from a hybrid H125S x R998; in which, H125S is a TGMS line imported from China; R998 is a pure line of VNUA, Vietnam
E30S	Selected from a hybrid E15S x IRBB21; in which, IRBB21 is a NIL lines bringing gene resistant to Bacterial Blight disease, origins from International Rice Research Institute (IRRI)

**Table 10:** Describing the data collecting methods on the investigated characters based on the Standard Evaluation System for Rice [1].

				Applied to	
Traits	Collection method	Data record		Parents	Hybrids
Growth stages of rice plants	Based on the development status, the life cycle of rice plants are separated into 9 growth stages	1	Germination	x	
		2	Seedling		
		3	Tillering		
		4	Stem elongation		
		5	Booting		
		6	Heading		
		7	Milk stage		
		8	Dough stage		
		9	Mature grain		

Maturity	Observing number of days from sowing to grain ripening (85% of grain on panicle are mature) at growth stage 9	Actual observed data		X	
Plant height	Measuring actual height (cm) from soil surface to tip of the tallest panicle at growth stage 7- 9. Record in whole numbers (without decimals)	1	Semidwarf (<110 cm)	X	
		5	Intermediate (110–130 cm)		
		9	Tall (>130 cm)		
Culm angle	Measuring the angle of the outmost tiller from the main culm at the full heading period (growth stage 7–9)	1	Erect (<30°)	X	
		3	Intermediate (~45°)		
		5	Open (~60°)		
		7	Spreading (>60°)		
		9	Procumbent		

Leaf blade color	Observing at growth stage 4- 6	1	Light green	X	
		2	Green		
		3	Dark green		
		4	Purple tips		
		5	Purple margins		
		6	Purple blotch		
		7	Purple		
Flag leaf angle	Measuring the angle of attachment between flag leaf blade and the main panicle axis at growth stage 4–5	1	Erect	X	
		3	Intermediate		
		5	Horizontal		
		7	Descending		

Brown rice shape	Measuring the length-width ratio of brown rice at growth stage 9 (after dehulling)	1	Slender (>3.0)	X	
		3	Medium (2.1–3.0)		
		5	Bold (1.1–2.0)		
		9	Round (<1.1)		
Pollen sterility	Collecting pollen from 10 flowers/plant, staining pollen grain with 1% Iodine Potassium Iodide (IKI) solution and observing under microscope under magnification 10×10, measuring the rate of sterile pollen	1	Completely sterile (100%)	X	
		3	Highly sterile (99.0–99.9%)		
		5	Sterile (95.0–98.9%)		
		7	Partially sterile (70.0–94.9%)		
		9	Partially fertile to fertile (<70%)		

Male sterility group	Based on the nature genetics of sterility	1 2 3 4 5 6	CMS	X	
			TGMS		
			PGMS		
			TPGMS		
			Transgenic type		
			Nuclear type		
Stigma exsertion	Measuring the percentage of florets (which have completed anthesis on a given days) showing exserted stigma on one or both side at stage 6–7	1	>70%	X	
		3	41–70%		
		5	21–40%		
		7	11–20%		
		9	0–10%		

Number of panicles/plant	Counting the average number of panicles containing filled grain on 10 random plants	Actual average data		X	X
Proportion of filled grain	Counting the average proportion of filled grain on all panicles of 10 random plants	Actual average data		X	X
Number of grains/panicle	Counting the average number of filled grain on all panicles of 10 random plants	Actual average data		X	X
1000-grain weight (g)	Measuring in grams of 1000 well- developed whole grain at growth stage 9, dried to 13% moisture content, using precision balance, three samples per line/hybrid	Actual average data		X	X
Grain yield	Harvesting 5 m^2^/plot (without border rows) at growth stage 9, measuring in tons per hectare at 14% moisture	Actual average data			X
Proportion of husked grain	Measuring the percentage in weight of husked grain from 1-kg sampled grain, 3 samples/hybrid	Actual average data			X
Proportion of milled grain	Measuring the percentage in weight of milled grain from 1-kg sampled grain, 3 samples/hybrid	Actual average data			X
Proportion of whole grain	Measuring the percentage of whole from 1-kg sampled grain after milling, 3 samples/hybrid	Actual average data			X
White grain length	Collecting random 10 white grains (after milling)/ sample, 3 samples/hybrid; measuring the grain length by precision calipers	Actual average data			X
White grain width	Collecting random 10 white grains (after milling)/ sample, 3 samples/hybrid; measuring the grain width by precision calipers	Actual average data			X

## 2 Experimental Design, Materials and Methods

### 2.1 Plant materials and cultivation

The experimental materials in this study comprised of seven male (Pollen Restorer) rice lines, four female (Thermo-Sensitive Genic Sterile- TGMS) rice lines, and their 28 hybrids derived from a Line x Tester (7 × 4) mating system. The name and origins of parental lines were described in Table 9. The parental lines were grown in summer-autumn 2017 to generate with agronomic traits that were characterized in winter-spring 2017–2018. The hybrids were grown in summer-autumn 2018 to evaluate the combining ability of parental lines.

All experiments were conducted at Crops Research and Development Institute (CRDI) of the Vietnam National University of Agriculture, Hanoi, Vietnam. In winter-spring 2017–2018, seeds of eleven parental lines were sown on 25 December 2017. After 25 days, the seedlings (with 5.5 leaves) were transplanted in a completely randomized design (CRD) without replication as described previously [2]. In summer-autumn 2018, seeds of 28 hybrids were sown on 20 June 2018. The 18-day old seedlings were transplanted in a randomized complete block design (RCBD) with three replications. Each experimental plot area was 15 m^2^; the growing density was 40 plants /m^2^. The agricultural practices applied for rice on the open field, including field preparation, fertilization, irrigation, pest and diseases managements were followed in accordance with National Technical Regulation on Testing for Value of Cultivation and Use of rice varieties (QCVN 01–55: 2011/BNNPTNT) issued by Ministry of Agriculture and Rural Development of Vietnam [3].

### 2.2 Data collection

Fourteen traits were collected from parental lines while 28 hybrids were assessed on 10 characteristics for evaluating combing ability. The data collection was in accordance with practices of International Rice Research Institute [1], the detailed description of data collection was displayed in Table 10.

### 2.3 Data analysis

The data analysis was conducted on the “Variance analysis LINE x TESTER Ver. 2.0″ software following the Line x Tester analysis method proposed by Kemphthorne [4,5].

#### 2.3.1 Variance analysis

The variation of GCA and SCA were calculated basing on the variation of GCA of line and tester

$$\sigma^{2}\mathrm{GCA}=\;\frac{l\left(\mathrm{Msl}-\mathrm{Mse}\right)+t\left(\mathrm{Mst}-\mathrm{Mse}\right)}{r\left(l+t\right)}$$

$$\sigma^{2}\mathrm{SCA}\;=\;\frac{\mathrm{Mst}*l-\mathrm{Mse}}{r}$$

In which, Msl: mean square of GCA line; Mst: mean square of GCA tester; Mse: mean square of error; Mst*l: mean square of SCA line x tester; l, t, and r: number of line, tester, and replication, respectively; σ^2^GCA: variance of GCA; σ^2^SCA: variance of SCA.

#### 2.3.2 Heritability analysis

The narrow sense heritability formula was estimated basing on the assumption that the total variation among hybrids equals to twice the GCA variance plus SCA variance while the genetic variance is equivalent to the variance of additive effects [6,7].

$$h^{2}\;=\;\frac{\sigma^{2}A}{\sigma^{2}P}\;=\;\frac{\sigma^{2}A}{\sigma^{2}A+\;\sigma^{2}D}=\;\frac{2\sigma^{2}\mathrm{GCA}}{2\sigma^{2}\mathrm{GCA}+\;\sigma^{2}\mathrm{SCA}}$$

In which, the additive variance: σ^2^A = 2.σ^2^GCA; the dominant variance: σ^2^D = σ^2^SCA

#### 2.3.3 Combining ability analysis

The contribution of combining ability effect on the morphological value was based on the models of Singh (1977) [8]:

$$Y_{\mathrm{ijk}}=\mu +g_{i}+g_{j}+S_{\mathrm{ij}}+e_{\mathrm{ijk}}$$

In which, Y_ijk_: value of hybrid between i^th^ line and j^th^ tester in k^th^ replication; μ: the average value of all hybrids in all replication (the general mean value); g_i_: general combining ability (GCA) of i^th^ line; g_j_: general combining ability (GCA) of i^th^ tester; S^ij^: specific combining ability (SCA) between i^th^ line and j^th^ tester; e_ijk_: error (environmental and replication effects to the (ijk)^th^ individual).

The combining ability data was estimated by the following formulas [8]:

$$\text{GCA}\;\text{of}\;i^{\text{th}}\;\text{line}:\;\;\;\;g_{i}=\;\frac{Y_{i..}}{\mathrm{tr}}\;-\;\mu$$

$$\text{GCA}\;\text{of}\;j^{\text{th}}\;\text{tester}:\;\;\;\;g_{j}\;=\;\frac{Y_{.j.}}{\mathrm{lr}}\;-\;\mu$$

$$\text{SCA}\;\text{between}\;i^{\text{th}}\;\text{line}\;\text{and}\;j^{\text{th}}\;\text{tester}:\;\;\;\;S_{\mathrm{ij}}\;=\;\frac{Y_{\mathrm{ij}.}}{r}\;-g_{i}-g_{j}-\mu$$

Whereby Y_i.._: sum value of hybrids between i^th^ line and all testers; Y_.j._: sum value of hybrids between j^th^ tester and all lines; Y_ij._: sum value of hybrids between i^th^ line and j^th^ tester in all replications; g_i_: general combining ability (GCA) of i^th^ line; g_j_: general combining ability (GCA) of i^th^ tester; μ: the average value of all hybrids in all replication (the general mean value); t: the number of testers; l: the number of lines; r: the number of replication.

#### 2.3.4 Estimating the contribution of parental lines to genetic variance of hybrid's traits

The contribution of line, tester, and line x tester towards the genetic variance of each character in F_1_ was calculated from the value of mean square of GCA line, GCA tester, and SCA line * tester as follows [4]:

$$\text{Contribution}\;\text{of}\;\text{line}\;\left(\text{male}\;\text{parent}\right)\;\left(\%\right)\;=\;\frac{Msl}{Msl+Mst+Msl*t}\;\times \;100$$

$$\text{Contribution}\;\text{of}\;\text{tester}\;\left(\text{female}\;\text{parent}\right)\;\left(\%\right)\;=\;\frac{Mst}{Msl+Mst+Msl*t}\;\times \;100$$

$$\text{Contribution}\;\text{of}\;\text{line}\;*\;\text{tester}\;\left(\text{interaction}\right)\;\left(\%\right)\;=\;\frac{Msl*t}{Msl+Mst+Msl*t}\;\times \;100$$

Where, Msl: mean square of GCA line; Mst: mean square of GCA tester; Msl*t: mean square of SCA line * tester

## Funding

The authors did not receive support from any organization for the submited work

## Ethics Approval

Not applicable

## CRediT Author Statement

**Quang Van Tran:** Conceptualization, Supervision, Resources, Methodology, Formal analysis, Validation, Writing – original draft, Writing – review & editing; **Long Thien Tran:** Formal analysis, Writing – original draft, Writing – review & editing; **Dung Thi Kim Nguyen:** Investigation, Data curtion; **Linh Hong Ta:** Conceptualization, Validation, Writing – review & editing; **Loc Van Nguyen:** Investigation, Data curtion, Writing – original draft, Writing – review & editing; **Tuan Thanh Nguyen:** Investigation, Data curtion, Validation, Writing – original draft, Writing – review & editing.

## Declaration of Competing Interest

The authors declare that they have no known competing financial interests of personal relationships that could have appeared to influence the work reported in this paper.

## Appendix Supplementary materials

Image, application 1
